# Supplementary material for: Towards a unified gating scheme for the CNBD ion channel family
Source: J Gen Physiol. 2025 Dec 11;158(1):e202513849. doi: 10.1085/jgp.202513849 (PMC12697245; doi:10.1085/jgp.202513849)
Supplement: Table S1 — shows unconstrained five-state gating polarity model parameters. [file jgp_202513849_tables1.docx]

**Table S1. Unconstrained five-state gating polarity model parameters.**

| Constructs | $K_{1}^{0}$ | $q_{1}$ | $K_{2}^{0}$ | $q_{2}$ | $K_{3}$ | $K_{4}$ |
| --- | --- | --- | --- | --- | --- | --- |
| D540K-Q664A hERG | 2.18 $\times$ 10^-5^ | -1.79 | 8.20 | 1.91 | 32.8 | 0.761 |
| D540K-L666A hERG | 1.63 $\times$ 10^-5^ | -2.63 | 1.55 $\times$ 10^-2^ | 1.67 | 0.204 | 34.4 |
| HHHEH | 9.56 $\times$ 10^-6^ | -1.37 | 1.17 | 1 | 299 | 2.21 |
| HHHEH_2_ | 8.31 $\times$ 10^-5^ | -1.44 | 17.2 | 1 | 44.7 | 0.126 |
| HHHES | 1.93 $\times$ 10^-4^ | -1.51 | 1.03 | 1.41 | 35.4 | 0.152 |
| HHHER | 9.85 $\times$ 10^-5^ | -1.30 | 9.40 | 2.67 | 89.3 | 0.193 |
| HHHEK | 17.55 | -1.05 | 0.812 | 1.32 | 0.0485 | 65.7 |
| HHHEA | 5.301 | -1.02 | 0.391 | 1 | 0.0682 | 31.9 |

See **Materials and Methods** for constraints and constants used to solve parameter values.
