# Supplementary material for: Towards a unified gating scheme for the CNBD ion channel family
Source: J Gen Physiol. 2025 Dec 11;158(1):e202513849. doi: 10.1085/jgp.202513849 (PMC12697245; doi:10.1085/jgp.202513849)
Supplement: Table S2 — shows five-state gating polarity model parameters. [file jgp_202513849_tables2.docx]

**Table S2. Five-state gating polarity model parameters.**

| Constructs | $K_{1}^{0}$ | $q_{1}$ | $K_{2}^{0}$ | $q_{2}$ | $K_{3}$ | $K_{4}$ |
| --- | --- | --- | --- | --- | --- | --- |
| HHHEH | 8.83 $\times$ 10^-6^ | -1.43 | 0.933 | 0.894 | 251 | 2.39 |
| HHHEH_2_ | 8.83 $\times$ 10^-6^ | -1.43 | 0.933 | 0.894 | 355 | 0.196 |
| HHHES | 8.83 $\times$ 10^-6^ | -1.43 | 0.933 | 0.894 | 987 | 0.162 |
| HHHER | 8.83 $\times$ 10^-6^ | -1.43 | 0.933 | 0.894 | 650 | 0.272 |

See **Materials and Methods** for constraints and constants used to solve parameter values.
