# Supplementary material for: Towards a unified gating scheme for the CNBD ion channel family
Source: J Gen Physiol. 2025 Dec 11;158(1):e202513849. doi: 10.1085/jgp.202513849 (PMC12697245; doi:10.1085/jgp.202513849)
Supplement: Table S3 — shows seven-state gating polarity model parameters. [file jgp_202513849_tables3.docx]

**Table S3. Seven-state gating polarity model parameters.**

| Constructs | $K_{1}^{0}$ | $q_{1}$ | $K_{2}^{0}$ | $q_{2}$ | $K_{3}$ | $K_{4}$ | $K_{5}$ | $K_{6}$ |
| --- | --- | --- | --- | --- | --- | --- | --- | --- |
| D540K hERG | 2.47 $\times$ 10^-6^ | -1.76 | 2.00 $\times$ 10^-2^ | 2.18 | 7.41 | 9.48 | 20.3 | 24.0 |
| D540K-Q664A hERG | 8.41 $\times$ 10^-7^ | -1.74 | 2.34 | 1.68 | 19.9 | 1.25 | 50.0 | 1.44 |
| D540K-L666A hERG | 1.82 $\times$ 10^-5^ | -2.45 | 4.67 $\times$ 10^-3^ | 1.63 | 0.896 | 0.562 | 0.436 | 200 |
| HHHEH | 2.39 $\times$ 10^-6^ | -1.47 | 0.342 | 0.892 | 17.9 | 1.80 | 43.5 | 3.68 |
| HHHEH_2_ | 2.39 $\times$ 10^-6^ | -1.47 | 0.342 | 0.892 | 25.0 | 11.2 | 47.3 | 0.159 |
| HHHES | 2.39 $\times$ 10^-6^ | -1.47 | 0.342 | 0.892 | 51.7 | 0.745 | 61.4 | 0.443 |
| HHHER | 2.39 $\times$ 10^-6^ | -1.47 | 0.342 | 0.892 | 40.0 | 2.69 | 51.1 | 0.345 |
| HHHEK | 2.39 $\times$ 10^-6^ | -1.47 | 0.342 | 0.892 | 2.08 $\times$ 10^6^ | 1.21 $\times$ 10^-4^ | 4.62 $\times$ 10^-2^ | 4.07 $\times$ 10^5^ |
| HHHEA | 2.39 $\times$ 10^-6^ | -1.47 | 0.342 | 0.892 | 2.08 $\times$ 10^6^ | 9.17 $\times$ 10^-5^ | 7.60 $\times$ 10^-2^ | 4.07 $\times$ 10^5^ |

See **Materials and Methods** for constraints and constants used to solve parameter values.
