# Supplementary material for: Towards a unified gating scheme for the CNBD ion channel family
Source: J Gen Physiol. 2025 Dec 11;158(1):e202513849. doi: 10.1085/jgp.202513849 (PMC12697245; doi:10.1085/jgp.202513849)
Supplement: Table S4 — shows six-state allosteric model parameters. [file jgp_202513849_tables4.docx]

**Table S4. Six-state allosteric model parameters.**

| Constructs | $K_{A}^{0}$ | $q_{A}$ | $K_{B}^{0}$ | $q_{B}$ | $\alpha$ | $\beta$ | $L$ |  |
| --- | --- | --- | --- | --- | --- | --- | --- | --- |
| HHHEH | 8.23 $\times$ 10^-6^ | -1.92 | 1.38 | 1.67 | 84 | 7.33 | 0.268 |  |
| HHHEK | 8.23 $\times$ 10^-6^ | -1.92 | 1.38 | 1.67 | 595 | 7990 | 5.11 $\times$ 10^-4^ |  |
| HHHEA | 8.23 $\times$ 10^-6^ | -1.92 | 1.38 | 1.67 | 681 | 7987 | 4.66 $\times$ 10^-4^ |  |

See **Materials and Methods** for constraints and constants used to solve parameter values.
