# Supplementary material for: Towards a unified gating scheme for the CNBD ion channel family
Source: J Gen Physiol. 2025 Dec 11;158(1):e202513849. doi: 10.1085/jgp.202513849 (PMC12697245; doi:10.1085/jgp.202513849)
Supplement: Table S5 — shows seven-state allosteric model parameters. [file jgp_202513849_tables5.docx]

**Table S5. Seven-state allosteric model parameters.**

| Constructs | $K_{A}^{0}$ | $q_{A}$ | $K_{B}^{0}$ | $q_{B}$ | $\gamma$ | $\delta$ | $M$ | $N$ |
| --- | --- | --- | --- | --- | --- | --- | --- | --- |
| HHHEH | 3.21 $\times$ 10^-6^ | -1.98 | 1.52 | 1.80 | 200 | 25 | 0.21 | 7.63 $\times$ 10^-2^ |
| HHHEK | 3.21 $\times$ 10^-6^ | -1.98 | 1.52 | 1.80 | 1224 | 509 | 3.54 $\times$ 10^-4^ | 8.07 $\times$ 10^-3^ |
| HHHEA | 3.21 $\times$ 10^-6^ | -1.98 | 1.52 | 1.80 | 1223 | 482 | 3.74 $\times$ 10^-4^ | 7.73 $\times$ 10^-3^ |

See **Materials and Methods** for constraints and constants used to solve parameter values.
